# ‘I’m adapting’: a qualitative study exploring how young adult migrants in the UK navigate asthma, environment, and healthcare

**DOI:** 10.1093/eurpub/ckag209

**Published:** 2026-11-10

**Authors:** Lucy Taylor, Laura Condon, Jennifer Creese, Bharathy Kumaravel

**Affiliations:** Leicester Real World Evidence Unit, Diabetes Research Centre, Leicester General Hospital, University of Leicester, Leicester, United Kingdom; Centre for Ethnic Health Research, Diabetes Research Centre, Leicester General Hospital, University of Leicester, Leicester, United Kingdom; School of Medical Sciences (Public Health & Epidemiology), University of Leicester, Leicester, United Kingdom; Leicestershire County Council, Leicester, United Kingdom; Leicester Medical School, University of Leicester, Leicester, United Kingdom

## Abstract

Migrants face barriers to healthcare, and young adults with asthma are at risk of poor outcomes. The intersection of migration, age, and deprivation is underexplored and may compound health inequalities in asthma care. This study explored the experiences of young adult migrants with asthma to identify barriers and facilitators to asthma management. A qualitative study was conducted in Leicestershire, UK. Migrants living in the UK for ≤5 years with asthma participated in semi-structured online interviews analysed using framework analysis. Seventeen participants aged 21–32 were recruited. They described substantial challenges in adjusting to the UK environment, including the impact of cold, damp housing, and unpredictable weather on asthma control, often leading to financial strain. Navigating healthcare systems was confusing and stressful, with delays in registration, prescriptions, and specialist referrals contributing to avoidable exacerbations and hospitalizations. Self-management strategies included layering clothing, monitoring weather forecasts, and seeking online information. Psychosocial burdens, including stigma around inhaler use, loss of social networks, and reduced autonomy, further undermined asthma control. Supportive General Practitioner (GP)–patient relationships and peer networks were important sources of support, although not all participants had access to them. Young adult migrants face multiple challenges in accessing healthcare and managing asthma after moving to the UK, even when they are confident in English. Better support to understand healthcare services and manage asthma in a new environment may help improve health outcomes. Future research should evaluate interventions that support access to healthcare and asthma management among migrant populations, including peer-support approaches.

## Introduction

Asthma affects 5.4 million people in the UK and remains a major cause of preventable morbidity, placing a substantial burden on individuals, primary care, and wider health services [1]. Effective management depends on sustained self-management and timely primary care access [2]. However, these requirements are not equally accessible across populations. Migrants, including refugees, asylum seekers, and undocumented individuals, face barriers to care that contribute to poorer asthma outcomes and persistent health inequalities [3–6].

Health inequalities in respiratory disease are recognized in national policy, including the NHS Long Term Plan, which highlights the impact of housing, air quality, and socioeconomic deprivation on health outcomes [7]. These policies recognize that asthma health outcomes are shaped by wider determinants such as housing quality, air pollution, employment, and access to care. Migrant and ethnic minority communities are disproportionately exposed to these determinants and may face additional challenges when accessing healthcare, including language barriers, differing expectations of healthcare systems, cultural beliefs about illness and treatment, and administrative obstacles such as difficulties registering with a GP or understanding referral pathways [6, 8, 9].

In Leicestershire, the local Joint Strategic Needs Assessment identified asthma as one of the most prevalent long-term conditions in deprived communities and noted that migrants face specific barriers to accessing care [10]. Migration has also been a driver of population growth within the county, and so understanding how migration-related experiences influence long-term condition management is important to improving health outcomes.

The aim of this study was to explore the experiences of recent migrants with asthma living in Leicestershire. We sought to understand how wider determinants of health influence asthma management, identify cultural and systemic barriers to accessing care, and generate evidence to inform locally relevant approaches to reducing inequalities in asthma outcomes.

### Theoretical/conceptual framework

Understanding health service use and navigation requires an interdisciplinary perspective, drawing on psychology (how individuals make decisions about health and make sense of their healthcare experiences), sociology (how social structures, resources, and expectations shape healthcare access), and anthropology (how cultural beliefs and understandings influence health-related behaviours). Cultural influences are particularly important in exploring migrant health-service usage: experiences are shaped by both migrants’ own beliefs about health and healthcare, and by the norms, values, and expectations embedded within the host healthcare system [11].

Health-service use in this study was informed by Yang and Hwang’s framework for understanding immigrant service utilization [12]. The framework proposes that health-service use is shaped by four domains: healthcare need (both self-perceived and professionally assessed); enabling resources (including financial, social support, and healthcare access); demographic factors (age, sex, genetic predisposition, health beliefs including cultural beliefs and adaptation into the host society, socioeconomic status including immigration status, which may influence healthcare entitlements and access); and macrostructural factors (such as healthcare policy, immigration policy, and wider political conditions).

This framework was selected because it recognizes that migrants’ healthcare use is shaped by factors extending beyond those typically considered in the general population. Transnational connections and previous experiences of healthcare systems may influence expectations of care, trust in healthcare professionals and institutions, help-seeking behaviour, and navigation of services in the host country. This perspective was particularly relevant to understanding how participants adapted to managing asthma within an unfamiliar healthcare system.

## Methods

A qualitative research design was deemed most appropriate because the study sought to explore migrants’ experiences, beliefs, and perceptions of asthma management and healthcare access. Semi-structured interviews were selected to facilitate in-depth exploration of these issues [13].

### Participants and sampling

Participants were recruited through self-selected sampling, via flyers and posters in workplaces and accommodation linked with migrant communities, supported by local authority colleagues. Interested individuals contacted the research team, received study information, and provided electronic informed consent following discussion to confirm understanding. Eligibility criteria were: migration to the UK within the last 5 years, residence in Leicestershire, and a diagnosis of asthma. Eligibility was assessed through participant self-report during the recruitment process and was not independently verified through medical records.

Recruitment continued until no substantially new codes or themes were identified in successive interviews. This assessment was made iteratively during data collection and analysis through discussion within the research team.

### Data collection

Interviews were conducted between March and April 2025. Participants were offered either face-to-face or online (Microsoft Teams), according to participant preference. All participants opted for online interviews. Individual interviews were chosen over focus groups to facilitate disclosure on potentially sensitive issues [14]. Interpreters were offered but not required.

Interviews were conducted by an experienced qualitative researcher (L.C.). A semi-structured topic guide, developed with public health colleagues and piloted in an earlier feasibility study, explored asthma management, environmental triggers, healthcare access, and self-management strategies. The guide was used flexibly, allowing participants to introduce issues important to them and enabling emerging topics to be explored in subsequent interviews. L.C. sought to establish rapport through a conversational interviewing style and by providing opportunities for participants to discuss issues they considered important. Interviews concluded when the topic guide had been covered and participants had no further experiences they wished to discuss.

Interviews were audio-recorded using Microsoft Teams. Automated transcripts were generated and subsequently reviewed against the audio recordings by L.C. to correct errors and ensure accuracy. Data were stored securely and will be retained for 3 years following study completion.

### Analysis

Data were analysed using framework analysis [15] supported by NVivo12. Four researchers (L.C., L.T., J.C., and B.K.) independently coded the same transcript and met to compare codes and develop an initial analytical framework. L.C. applied and refined this framework across the remaining transcripts, with emerging codes and interpretations discussed regularly within the research team. Codes were grouped into categories and subsequently developed into themes through iterative comparison across the dataset. Analysis was primarily inductive, with Yang and Hwang’s framework informing interpretation rather than acting as a deductive coding structure.

### Ethics

This research study was reviewed and approved by the University of Leicester (Ref. 2025-2100-4907). Additional ethical care included ensuring that the interviewer was respectful of the participants’ cultural background and beliefs, and being particularly sensitive to any emotional responses relating to their experience of having migrant status [16]. All participants received a £20 voucher as compensation for their time.

## Results

Seventeen participants (10 females, 7 males; aged 21–32, median 25) were interviewed. They had migrated between 18 months and 5 years prior, from countries across Africa, Asia, the Americas, and Oceania.

Table 1 presents the demographic information and pseudonyms for each participant. All names are pseudonyms assigned by the research group in consultation with the participant.

**Table 1:** Demographic details of participants (*n* = 17).

Pseudonym	Gender	Age	Country of origin	Time since migration
Adeyimi	F	Did not disclose	South Africa	4 years
Aziz	M	Did not disclose	Malaysia	2 years
Chinwe	F	22	Ghana	4 years
Desta	F	21	Nigeria	18 months
Emma	F	23	USA	3 years
Faisal	M	28	Nigeria	3 years
Halimah	F	25	Nigeria	3 years
Idowu	F	23	Nigeria	2 years
Jeoma	F	25	Nigeria	2 years
Kele	F	28	Kenya	3 years
Lishan	M	27	Kenya	18 months
Max	M	29	Australia	5 years
Neryse	F	22	Jamaica	18 months
Paul	M	32	South Africa	5 years
Seyna	F	24	Nigeria	3 years
Wasim	M	22	Egypt	2 years
Yvette	F	28	Canada	4 years

Framework analysis identified four overarching themes: adapting to the UK environment and asthma triggers; navigating the NHS and barriers to care; self-management and health information sources; and psychosocial impact and support networks.

### Adapting to the UK environment and asthma triggers

Moving to the UK often meant learning to manage asthma in unfamiliar and difficult conditions. Participants described being unprepared for the often colder, damper climate, and the effect it had on their symptoms. Some also identified unfamiliar environmental triggers, including dust, cooking fumes, and spices, which they had not previously associated with worsening asthma. Seyna remembered her first accommodation: ‘It was very cold and the walls were damp’, which she recalled immediately worsened her asthma. Others spoke about how the unpredictable weather meant they had to change their routines, such as checking weather forecasts and avoiding exposure to cold weather.

Many developed strategies to cope, such as layering clothing or using heating. As Seyna described, ‘I’ve learned to cover myself better and expose myself less to direct cold’. Waso recalled how her family helped her adjust: ‘Mum got me a lot of coats and we have a fireplace, so I’m always in my coat. I’m adapting’. For Chinwe, however, it meant feeling confined even indoors: ‘I’m completely covered up, even my hands and feet, because I don’t want that direct contact with the cold’. These strategies often came at a cost. Desta explained that using heating constantly brought ‘a lot of financial stress’.

Several participants reflected that clearer guidance at the point of arrival to the UK could have reduced the anxiety of this transition. They suggested tailored education about environmental triggers, including damp or dusty housing, cold weather, and cooking fumes, alongside practical advice on recognizing triggers, avoiding exposure where possible, and managing symptoms in the UK climate.

### Navigating the NHS and barriers to care

For many, making sense of the NHS was a great challenge. Registering with a GP, securing prescriptions, or arranging specialist care felt confusing and emotionally draining, particularly when participants experienced delays in treatment or worsening symptoms while trying to access support. Paul described the experience simply: ‘I had some challenges which I feel like were not really nice trying to access healthcare here’.

Jeoma remembered feeling overwhelmed when she first arrived in the UK: ‘I didn’t know how to register with my GP or access specialist care … I had to wait several weeks for my prescription to be approved. During this time I experienced several severe symptoms, including hospitalisations’. Others described hospital visits as impersonal, while long waits for both primary and out-of-hours services created uncertainty. Adeyimi recalled calling the out-of-hours GP line only to receive a callback ‘maybe seven hours later’.

Some felt lost in the complexity of the system. Yvette explained, ‘There are so many branches of healthcare … I can be lost. And because I don’t have insurance, it’s quite expensive’. Participants suggested clearer information on how to register with a GP, access prescriptions, and navigate urgent and specialist care after arriving in the UK, alongside more flexible appointments and training for reception, administrative, and clinical staff to support migrants in navigating the NHS.

### Self-management and health information sources

Despite these barriers, most participants described developing their own self-management routines. These included avoiding irritants, keeping inhalers close, and monitoring the weather. Faisal explained how he incorporated the weather forecast into his daily planning: ‘Here it’s more about moisture and feeling it, so I started checking the weather report almost every day. It helps me know when I need to be extra careful’.

However, participants noted that new and unfamiliar environmental triggers, especially damp housing, made self-management difficult. Desta reflected, ‘… it sometimes feels like I’m in a damp area, so that makes me feel uncomfortable and makes my health feel like I’m about to crash. So I have this heater, and that causes a lot of financial stress’. Chinwe described the learning process: ‘It took a lot for me to understand why it was happening so often. Trigger awareness is very important, especially because the environment is different from home. We need that awareness of how to care for ourselves here’.

Participants drew on a range of sources when seeking information about asthma, including healthcare professionals and the internet. Participants valued healthcare professionals who showed interest in their condition and were viewed as trusted sources of advice. Kele described her GP as ‘invested in my health … really interested in me having good healthcare here’. Others turned to online sources because they were readily accessible. Emma said the ‘fastest thing I do is search online’. However, participants were often cautious about relying on online information alone. As Kele explained: ‘I have actually tried looking on the Internet for information in the past and I’d say there is a whole lot of information there and a lot of them are mixed up …’ Neryse similarly reflected that online information ‘could offer useful information or not. You just got to see if it’s true’.

Participants also described a need for additional support in managing asthma after moving to the UK. They highlighted a need for tailored educational resources to support understanding of environmental triggers, healthcare navigation, and asthma self-management in the UK context. Seyna reflected that ‘it took a while for me to realize the damp rooms were giving me problems … If I’d had this information much earlier, I could have taken steps to prevent the repeated asthma attacks myself’. Idowu similarly suggested that ‘there could be more information on how the NHS operates, especially to new people … we do not know where to go. We do not know the right things to do, so it’s just a struggle’. Participants also highlighted the importance of recognizing differences in health beliefs and previous healthcare experiences when providing asthma education after migration. As Idowu explained, ‘there were cultural nuances … different attitudes towards medical interventions … healthcare can vary. There’s this issue of the standard healthcare system not really agreeing with traditional healthcare … We can work together to make healthcare better’.

### Psychosocial impact and support networks

Asthma carried a significant psychosocial burden for many participants. Some described stigma, embarrassment, and a sense of isolation. Lishan explained, ‘I don’t like people to pity me … it makes me look like someone who doesn’t take care of myself’. Desta recalled that after one severe episode, ‘some people didn’t want to talk to me again; they avoided me’.

Avoiding triggers often limited social participation. Adeyimi’s friends frequently asked why she went home early, but as she explained, ‘At home I have a little bit of control … It kind of infringes on my social life’. Aziz reflected on the impact on his identity: ‘I’m not pushing myself to extremes, and that sometimes makes me feel less of myself. I’m having a limited life now because most of the things I’m passionate about, I’m just not allowed to do’.

At the same time, social networks were described as vital sources of support. Kele relied on a friend who not only made meals but also accompanied her to appointments: ‘She actually went with me to see my GP’. Others described the difficulty of losing these supports after moving or when friends relocated. ‘I had a friend once that I could call any time, but then she moved away’, said Adeyimi.

Participants felt that opportunities to connect with others who shared similar experiences of asthma and migration could help reduce feelings of isolation and provide mutual encouragement. As Paul suggested: ‘If there’s a medium where people like me who have asthma could come together, even if it’s online, we could share our experiences and solutions’. Faisal explained that joining an asthma support group helped because ‘sharing my experience and hearing from other people’s experiences gives me new ideas’.

## Discussion

### Summary

This study highlights the compounded challenges faced by young adult migrants with asthma in a deprived UK setting. Even among English-fluent and relatively health-literate participants, NHS systems proved difficult to navigate.

### Strengths and limitations

This study’s strengths lie in its focus on a high-risk group: young adult migrants, a demographic shown to have higher asthma prevalence, poorer control, and above-average hospital admissions [1]. Grounding the project in a local Health Needs Assessment ensured the research questions were driven by community need, enhancing the relevance of findings to local service planning. The inclusion of participants from multiple world regions enabled exploration of barriers that were shared across different migrant groups.

Several limitations should be considered. As a qualitative study conducted in a single geographical area, the findings may not be transferable to all migrant populations or healthcare settings. Although interpreters were offered, all participants were sufficiently confident in English to participate without one. Consequently, the experiences of migrants with greater language barriers may be underrepresented. Recruitment through workplaces and accommodation settings may have limited participation from more marginalized groups, such as asylum seekers or undocumented migrants, who may experience even greater barriers [4].

### Comparison with existing literature

Our findings echo wider evidence that migrants in the UK face fragmented pathways, unclear entitlement, and perceived discrimination [4, 17]. They also align with asthma research in minority ethnic communities that highlights challenges in accessing supported self-management and asthma care [6]. While ethnic inequalities in healthcare are well documented [18], participants attributed difficulties accessing care to navigating an unfamiliar healthcare system following migration. This suggests that migration-related experiences may contribute to barriers to care alongside ethnicity.

Young adult migrants in the UK face a complex set of challenges that undermine effective asthma management. Participants in our study described how cold, damp housing exacerbated symptoms while simultaneously creating financial strain through heating costs, echoing wider evidence on housing, deprivation, and respiratory health [3, 19]. Housing has been identified as a critical social determinant of health and wellbeing for migrant and refugee populations across high-income countries, with housing impacting not only physical but mental health outcomes [20].

Self-management strategies were often adaptive responses to unfamiliar environmental challenges. Participants monitored weather forecasts, modified daily routines, and sought information from both healthcare professionals and online sources to manage asthma in a new environment. While online information was readily accessible, participants frequently expressed uncertainty about its reliability and generally placed greater trust in healthcare professionals when making decisions about their asthma. This reflects wider evidence that migrants often draw on multiple sources of information when seeking health advice [21]. Many participants also described a need for more tailored education about environmental triggers, healthcare navigation, and asthma management in the UK context. Participants also valued opportunities to learn from others with lived experience of asthma. Structured peer-support interventions have shown benefits for asthma self-management in other populations [22], suggesting that similar approaches may warrant further evaluation among migrant populations.

Psychosocial burdens were striking. Stigma around inhaler use, the loss of familiar social networks, and restrictions on lifestyle created feelings of isolation and reduced autonomy. Previous work has highlighted the importance of social and cultural influences on asthma management [3, 9]. Our findings extend this literature by demonstrating how migration can disrupt social support, belonging, and self-management, echoing wider evidence that changes in social relationships and sense of belonging following migration can have important consequences for health and wellbeing [23, 24]. Social networks and social capital have been identified as important influences on self-management of long-term conditions [25], and disruption of these networks following migration may therefore present an additional barrier to effective self-management.

Our findings build on UK studies of asthma in migrant children and South Asian communities [7, 9, 26, 27] by highlighting similar and in some cases, intensified barriers in recently arrived, diverse migrant groups. Comparable challenges have been documented in the UK more broadly, where new migrants often struggle with GP registration, continuity, and culturally sensitive care [28]. International comparisons also underline the systemic nature of these issues: migrants in Canada and Australia [29, 30] report similar problems of fragmented pathways and poor cultural adaptation of services. These countries all treat migrant patients within the context of state-run healthcare services similar to the UK’s NHS; additional healthcare policy factors, such as insurance and out-of-pocket costs raised by some participants in this study, have been noted as significant barriers to effective asthma care for migrants in privatized healthcare systems like the USA [31], or in state-run systems like France where new migrants must purchase private health insurance to cover their initial residence [32]. By situating asthma within this wider migration and health literature, our findings demonstrate that system navigation challenges persist even when language and literacy barriers are absent.

While some participants described supportive GP relationships, others encountered confusion, long waits, and limited continuity of care. This suggests that the variability of migrant experiences may contribute to inequalities in asthma control. This disparity mirrors broader observations that primary care often lacks structured approaches for engaging recently arrived migrant populations [28, 33]. Future research should evaluate how primary care can systematically identify new migrants at risk of poor outcomes and test strategies to promote trust, continuity, and self-management support. The potential for lay-led or peer support interventions, in partnership with migrant communities, in bridging gaps left by conventional care models and improving equitable access to asthma management should also be explored.

At a policy level, our findings underline the importance of considering migrant populations in local health needs assessments and in national strategies for long-term condition management. Collaborations with community organizations and peer networks may also represent a promising area for research, given the role of informal support in bridging gaps left by formal healthcare.

### Implications for practice, policy, and research

Our findings suggest that migrants may require additional support navigating healthcare systems even when language barriers are absent. Primary care teams should consider providing clearer information about registration, referral pathways, prescriptions, and urgent care access, alongside asthma education that addresses environmental triggers commonly encountered in the UK.

At a policy level, our findings highlight the importance of considering migrant populations within local health needs assessments and long-term condition strategies. The findings also highlight the importance of addressing wider determinants of health, including housing quality and socioeconomic disadvantage.

Future research should evaluate interventions that support asthma self-management and healthcare navigation among migrant populations, including peer-support approaches delivered by individuals with lived experience of asthma and partnerships with community organizations that support migrant communities. Further work is also needed to explore the experiences of migrants with asthma who face greater language barriers or more insecure social circumstances.

## Data Availability

Data are available from the authors upon reasonable request.
